# The association between growth differentiation factor-15, erythroferrone, and iron status in thalassemic patients

**DOI:** 10.1038/s41390-023-02729-5

**Published:** 2023-07-18

**Authors:** Ilham Youssry, Rania M. Samy, Mohamed AbdelMohsen, Niveen M. Salama

**Affiliations:** 1 Pediatric Department, Faculty of Medicine, Cairo University, Cairo, Egypt; 2 Clinical Pathology Department, Faculty of Medicine, Cairo University, Cairo, Egypt

## Abstract

**Background:**

Iron overload can result in grave consequences in thalassemic patients, despite the availability of iron chelators. Therefore, alternative pathways aiming to reduce iron toxicity are currently investigated. Among which, reduction of iron absorption through control of hepcidin production appears to be promising. In this study, we investigated growth differentiation factor-15 (GDF15) and erythroferrone (ERFE) as potential suppressors of hepcidin.

**Methods:**

This cross-sectional study was conducted on 61 thalassemic patients and 60 healthy controls. The frequency of GDF15 gene polymorphism (rs4808793) (-3148C/G), serum level of GDF15 and erythroferrone were measured and correlated with those of hepcidin and serum ferritin.

**Results:**

The presence of GDF15 gene mutations were significantly higher in the patients’ group compared to controls (*P* value 0.035). Also, thalassemia patients had significantly higher levels of GDF15 and ERFE and lower hepcidin levels than controls (*P* value < 0.001). Serum hepcidin level showed significantly negative correlations with GDF15, ERFE, reticulocyte count, LDH level, and serum ferritin. Contrarily, it had highly significant positive correlation with hemoglobin.

**Conclusions:**

High level of GDF15 and/or ERFE may inhibit hepcidin production and increase iron load in patients with thalassemia; therefore, medications that suppress their actions may provide new therapeutic potentials for iron toxicity.

**Impact:**

- Iron overload continues to be a major contributor to high morbidity and mortality in patients with thalassemia.
- New strategies together with proper chelation, need to be developed to minimize the effect of iron toxicity.
- Growth differentiation factor-15 (GDF15) and erythroferrone (ERFE) inhibit hepcidin production and increase iron levels in conditions with ineffective erythropoiesis.
- Medications that suppress the production or interfere with the action of GDF15 or ERFE may represent new therapeutic potentials for iron toxicity.
- Prevention of iron toxicity will significantly reduce morbidity and mortality and improve the quality of life of thalassemia patients.

## Introduction

In conditions with ineffective erythropoiesis, elevated tissue iron storage continues to be a serious sequalae leading to high morbidity and mortality despite the availability of different chelators.^1^ Hence the need to understand the underlying mechanisms and to search for alternative strategies to minimize iron overload and toxicity.

Hepcidin suppresses the release of iron by combining with ferroprotein gate in enterocyte, hepatocyte and macrophages. this triggers the endocytosis of both molecules, with consequent lysosomal degradation.^2^ Growth differentiation factor-15 (GDF15) and erythroferrone (ERFE) are suggested to play a role as inhibitory mediators of hepcidin production.^3^

GDF15 was suspected to play a role in the process of cell maturation and controlled death.^4^ The human GDF15 is encoded by a gene located on chromosome 19, in which eight polymorphisms were proved to regulate its serum level.^5^ Among those polymorphisms, rs4808793 that results from C allele replacing G allele (-3148C/G), was associated with the highest GDF15 expression.^6^ The high GDF15 levels in cases with ineffective erythropoiesis, especially thalassemia, were suggested to be involved in the pathological iron burden via incomplete hepcidin inhibition.^4^

ERFE is now considered the main hepcidin downstream regulator^7^ through suppression of bone morphogenetic protein (BMP) pathway in the liver.^8^ ERFE, therefore, increases iron absorption from the intestine, mobilizes iron from its’ stores, and increases iron available for hemoglobin synthesis in situations with stress erythropoiesis.^9^ Elevated level of ERFE may result from erythropoietin (EPO) hormone stimulation that results in the formation of huge amounts of immature red cells that secrete ERFE with subsequent inhibition of hepcidin.^10^ Following blood transfusions, the serum level of ERFE decreases temporarily, while serum hepcidin levels go up, therefore medications that suppress the action of ERFE may be crucial in controlling the iron burden in conditions with ineffective erythropoiesis.^11^

In our study, we aim to investigate the serum level of GDF15, ERFE, and hepcidin, as well as to assess the frequency of GDF15 gene polymorphism (rs4808793) (-3148C/G) in a group of transfusion-dependent thalassemia patients. The study also aims at assessing the role of all studied variables in iron overload in this group of patients.

## Patients and methods

This cross-sectional study was performed on 61 transfusion-dependent thalassemia major patients (36 males and 25 females), who were diagnosed and following in the Hematology Clinic, New Children Hospital, Cairo University. The study protocol was approved by the ethical committee of Cairo University.

Convenient thalassemia major patients’ sample, for all patients who attended the Hematology clinic on Tuesdays between 1 June 2021 and 31 August 2021 were enrolled in the study Patients who received blood within the previous 4 weeks or had suspected concurrent infections or organ dysfunction were excluded from the study. Sixty age and sex-matched healthy children were recruited from the out-patient clinic of the same hospital over the same period of time and were enrolled as a control group. Written informed consents was obtained from all participants and/or their legal guardians.

All patients were subjected to complete history taking, full clinical examination, routine laboratory testing (CBC, reticulocyte count, LDH level, bilirubin total and direct, Hb electrophoresis, and serum ferritin level). All patients and controls were tested for the serum level of hepcidin, GDF15 and ERFE. Also, the frequency of GDF15 gene polymorphism (rs4808793) (-3148C/G) was evaluated.

### Genotyping method

Two ml of blood were collected on EDTA containers and were stored at −20 °C and were used for DNA extraction using Gene JET™ Genomic DNA Purification Kit (Thermo Scientific, catalog number: #K0781). Extracted DNA was stored at temperature between −20 and −70 °C. GDF15 gene polymorphisms (rs4808793) (-3148C/G) were studied using polymerase chain reaction–restriction fragment length polymorphism (PCR-RFLP) based on the method described by Tagu and Moussard.^12^

Serum GDF15 detection was done by enzyme linked immune-sorbent assay (ELISA).^13^ We used ELISA Kit (Cat. No: E0037Hu, Bioassay laboratory technology, China) as well as Human Protein FAM132B (FAM132B) (Cat.No: E3957Hu, Bioassay Laboratory Technology, China). Serum hepcidin was also estimated by ELISA (Cat.No: CEB979Hu, Bioassay Laboratory Technology, China).

### Statistical methods

The collected data was coded, processed, and analyzed using the SPSS (Statistical Package for Social Sciences) version 26 for Windows® (IBM SPSS Inc, Chicago, IL). Data were tested for normal distribution using the Shapiro–Wilk test. Numbers and percentages were used for descriptive statistics of qualitative variables while mean and standard deviation were used for quantitative ones. Chi-square and Fisher’s Exact tests were used to test significance for qualitative variables. As for quantitative variables, independent sample *t* test and one-way ANOVA were used for normally distributed variables, while Mann–Whitney and Kruskal–Wallis tests were used for variables that were not normally distributed. Correlation was used to test the relation between two quantitative variables. Diagnostic performance was done using ROC curve. *P* value was considered statistically significant if less than 0.05.

## Results

Our study included 61 transfusion-dependent thalassemia patients (36 males and 25 females), their ages ranged between 6 and 21 years [median (IQR) 13 (11–17)] and the male to female ratio was 1.5:1. Consanguinity was positive in 50.8% of the patients. The mean age at diagnosis was 7.74 ± 3.17 months [median (IQR) 7 (6–10) with range 4–16 months], the mean age at the transfusion was 7.74 ± 3.17 months (range 4–16 ms) with 77% of patients receiving monthly transfusion. The mean duration of transfusion was 12.72 ± 4.35 years (range 6–20 years), and the mean volume of transfusion was 160.08 ± 38.99 cc/kg/year. The mean duration of chelation therapy was 4.20 ± 1.63 years. In this study, the median of serum ferritin was 1094 (353.2–2060.4) ng/ml, serum ferritin was <1000 in 44.3% of the patients, and >1000 in 55.7%.

Serum levels of GDF15 and ERFE were significantly higher while serum hepcidin level was significantly lower in patients’ group than in control group. Also, both homozygous and heterozygous GDF15 gene mutations were significantly higher in the patients’ group than in the control group (Table 1).

**Table 1:** Comparison between patient and control groups regarding GDF15, hepcidin, and erythroferrone.

		Control group No. = 60	Patients group No. = 61	*P* value
GDF15 by PCR	N (CC)	44 (73.3%)	32 (52.5%)	0.035
	HT (CG)	16 (26.7%)	27 (44.3%)	
	H (GG)	0 (0.0%)	2 (3.3%)	
GDF15 (ng/ml)	Median (IQR)	59 (35–98.1)	576.5 (182.6–1200.5)	<0.001
	Range	7.8–218	10.5–4842.4	
Erythroferrone (ng/ml)	Median (IQR)	159.6 (86.7–349.1)	992.4 (215–1571.4)	<0.001
	Range	15.2–5021	48.4–5803.8	
Hepcidin (ng/ml)	Median (IQR)	2130 (1139.2–5464)	535.7 (336.4–814.1)	<0.001
	Range	346.7–12,164.1	99.3–13,053.9	

*N* CC normal (wild type), *Ht* CG heterozygous, *H* GG homozygous, *GDF15* growth differentiation factor 15, *PCR* polymerase chain reaction, *SD* standard deviation, *IQR* interquartile range.

Twenty-nine (47.5%) of subjects in the patients’ group had GDF15 gene polymorphism, whether homozygous or heterozygous. The aforementioned patients had significantly higher serum level of GDF15 and ERFE, while significantly lower serum hepcidin level than in patients’ group with normal GDF15 genotype (Table 2).

**Table 2:** The association between the presence of GDF15 polymorphism and serum levels of GDF15, hepcidin, and erythroferrone.

		Cases (*N* = 61)		*P* value
		CC (*N* = 32)	CG + GG (*N* = 29)	
GDF15 (ng/ml)	Median (IQR)	252.15 (99.75–522.35)	1271.4 (737.6–1530.6)	<0.001
	Range	38.4–927.8	10.5–4842.4	
Erythroferrone (ng/ml)	Median (IQR)	464.75 (172.4–1270.5)	1388.6 (1005.7–1792.4)	0.002
	Range	48.4–3276.2	109.6–5803.8	
Hepcidin (ng/ml)	Median (IQR)	792.95 (618.5–1478.25)	327.9 (273.5–391.1)	<0.001
	Range	475.7–13,053.9	99.3–560.4	

*N* number, *CC* normal (wild type), *CG* heterozygous, *GG* homozygous, *GDF15* growth differentiation factor 15.

In this study, both serum ferritin and serum GDF15 levels were significantly higher in patients who underwent splenectomy than in those who did not, with *P* values of 0.042 and 0.036, respectively.

Our patients’ group was further divided into 2 subgroups based upon their ferritin level; higher ferritin group (≥1000 ng/ml, *N* = 34) and lower ferritin group (<1000 ng/ml, *N* = 27). Patients with higher ferritin levels had significantly higher frequency of GDF15 polymorphism with *P* value < 0.001, they also had significantly higher serum levels of GDF15, ERFE, and lower serum level of hepcidin (*P* value < 0.001 for all).

By correlating the studied variables, both GDF15 and ERFE showed highly significant positive correlation with each other and a significantly negative correlation with hepcidin (Table 3). The serum level of both GDF15 and ERFE had significantly negative correlations with Hb and significantly positive correlation with the reticulocyte count, LDH level, and serum ferritin. The serum level of hepcidin had significantly negative correlation with reticulocyte count, LDH level, serum ferritin and a significantly positive correlation with hemoglobin (Table 3). Statistically significant correlations were not found between either GDF15, hepcidin, or ERFE and all the other studied parameters (as weight, height, age of the patients, age at diagnosis, age at first transfusion, volume of transfusion/year, duration of chelation therapy, results of Hb electrophoresis, kidney or liver function tests)

**Table 3:** Correlation between GDF15, hepcidin, and erythroferrone with different studied parameters.

	GDF15 (ng/ml)		Hepcidin		Erythroferrone	
	*R*	*P* value	*R*	*P* value	*R*	*P* value
GDF15 (ng/ml)	–	–	**−0.574**	**<0.001**	**0.493**	**<0.001**
Hepcidin (ng/ml)	**−0.574**	**<0.001**	–	**–**	**−0.362**	**0.004**
Erythroferrone (ng/ml)	**0.493**	**<0.001**	**−0.362**	**0.004**	–	–
HbA %	−0.080	0.540	0.203	0.117	−0.071	0.588
HbA2 %	0.049	0.708	−0.003	0.985	0.059	0.652
HbF %	0.047	0.717	−0.169	0.192	0.023	0.859
Hb (g/dl)	−0.955**	**<0.001**	0.535**	**<0.001**	−0.410**	**0.001**
Platelet count (10^3^/cmm)	0.168	0.195	−0.210	0.105	−0.133	0.306
TLC (10^3^/cmm)	0.052	0.688	−0.184	0.156	0.005	0.968
Reticulocytes (%)	**0.940**	**<0.001**	**−0.593**	**<0.001**	**0.453**	**<0.001**
LDH level (U/I)	**0.952**	**<0.001**	**−0.533**	**<0.001**	**0.440**	**<0.001**
Serum ferritin (ng/ml)	**0.950**	**<0.001**	−0.616**	**<0.001**	**0.526**	**<0.001**
Serum Ca (mg/dl)	0.038	0.772	−0.079	0.547	0.023	0.860
Serum PO_4_ (mg/dl)	0.047	0.722	−0.134	0.302	0.114	0.381
ALP (U/l)	**−0.344**	**0.007**	0.152	0.242	−0.162	0.213

*Hb* hemoglobin, *TLC* total leukocyte count, *LDH* lactate dehydrogenase, *Ca* calcium, *PO*_*4*_ phosphate, *ALP* alkaline phosphatase.

Statistically significant values are in bold.

The ROC curve analysis for GDF15, hepcidin and ERFE among cases and control groups, for detection of iron overload, revealed the following: the abnormal cut-off point of GDF15 of >177.9 ng/ml and area under the curve (AUC) of 0.907 were selected to obtain a sensitivity of 77.05% and specificity of 98.33%. Regarding hepcidin, the abnormal cut-off point of ≤835.2 ng/ml, and AUC of 0.847 were selected with 77.05% sensitivity and 86.67% specificity. For ERFE, the abnormal cut-off point of >507 ng/ml, and AUC of 0.766 were selected with 62.30 % sensitivity and 85% specificity.

In this study, the serum ferritin was taken as an indicator of iron overload. Therefore, assessment of the serum ferritin levels according to the cut-off value of GDF15, ERFE, and hepcidin revealed that, significantly higher serum ferritin levels were found in patients with high serum levels of GDF15, high serum levels of ERFE and low serum levels of hepcidin (Table 4).

**Table 4:** Serum ferritin levels among cases according to the predetermined cut-off value for GDF15, erythroferrone, and hepcidin (total patients = 61).

		GDF15 < 177.9 ng/ml 14 patients	GDF15 ≥ 177.9 ng/ml 47 patients	*P* value
Serum ferritin ng/ml	Median (IQR)	239.00 (179–273)	1203.10 (612.00–2510.5)	<0.001
	Range	150–280	316.00–7986.05	
		**ERFE** < **507** **ng/ml**	**ERFE** ≥ **507** **ng/ml**	
		**23 patients**	**38 patients**	
Serum ferritin ng/ml	Median (IQR)	316.0 (258–544)	1212.6 (924.97–2612.27)	<0.001
	Range	150–3754.5	158.00–7986	
		**Hepcidin** < **835.2** **ng/ml**	**Hepcidin** ≥ **835.2** **ng/ml**	
		**47 patients**	**14 patients**	
Serum ferritin ng/ml	Median (IQR)	1203.1 (400.6–25,510.5)	434.5 (273.3–544)	0.006
	Range	150–7986	220–1131.7	

In this study, 34 patients (55.7%) had a serum ferritin level ≥1000 ng/dl. To study the distribution of these patients according to the serum level of GDF15, ERFE, and hepcidin, we found that all patients with a serum ferritin >1000 ng/dl had a serum GDF15 ≥ 177.9 ng/ml, 29 patients (85.29%) with a serum ferritin >1000 ng/dl had a serum ERFE ≥ 507 ng/ml, and 32 patients (94.11%) with a serum ferritin levels >1000 ng/ml had a serum hepcidin <835.2 ng/ml.

## Discussion

In this study, serum ferritin 1000 ng/ml was used as a cut-off to categorize the iron status of our patients, since the majority of guidelines use this value to depict the development of iron overload and the need to start chelation.^10^

### GDF15 polymorphism (rs4808793) (-3148C/G)

Both homozygous (G.G.) and heterozygous (C.G.) mutations were significantly higher in the patients’ than controls with *P* value of 0.035. To the best of our knowledge, no previous studies were conducted in Egypt nor the Mediterranean region to support our findings. The serum levels of GDF15 and ERFE were significantly higher, while that of hepcidin was significantly lower, in the presence of polymorphism “whether homozygous or heterozygous” when compared to those with normal genotype in thalassemia patients, with *P* values < 0.001, 0.002, and <0.001, respectively. Similar findings regarding GDF15 polymorphism and hepcidin were detected by other researchers.^3,6^

In this study, the serum level of GDF15 was significantly higher in thalassemia patients (median 576.5 ng/ml) than in control group (median 59 ng/ml) with a *P* value < 0.001. These results are in accordance with other studies.^14,15^ This could be explained by the fact that ineffective erythropoiesis and increased iron influxes occurring in thalassemia patients may produce signals with consequent stimulation of GDF15 gene expression which causes reduction of hepcidin levels. The reduced hepcidin levels thereby lead to increased intestinal iron absorption and aggravates the pre-existing iron overload.^16^

In the present study, GDF15 levels were significantly higher in splenectomised patients (median = 737.6 ng/ml and 495.85 ng/ml in non splenectomised) with a *P* value of 0.036. Similarly, studies on thalassemia intermedia^17^ and sickle cell anemia^18^ detected significantly higher levels of GDF15 in patients with the more severe form of the disease who underwent splenectomy. These findings could be explained by the fact that splenic reticuloendothelial cells are crucial in removing apoptotic and aging cells including red blood cells. Following splenectomy, the rate of intravascular hemolysis and vascular complications increases, resulting from a combination of hypercoagulable state, increased platelet activation, and dyslipidemia, with subsequent erythrocyte fragmentation and formation of a huge amount of GDF15.^19^

This study showed that GDF15 had a highly significant negative correlation with Hb and hepcidin and a highly significant positive correlations with EFRF, reticulocyte count, LDH level, and serum ferritin. Other study also showed statistically significant negative correlation with Hb.^17^ Porter and his colleagues, did not find any relationship between GDF15 and the patients' hemoglobin. However, it is noteworthy to mention that they targeted non-transfusion-dependent thalassemia patients in their study.^20^ Similarly previous studies found a statistically significant negative correlation between GDF15 and hepcidin in thalassemia patients.^6,15^ In accordance with our results, Athiyarath and colleagues also found a highly significant positive correlation between GDF15 level and serum ferritin.^6^ In another study conducted in thalassemia intermedia patients, significant positive correlations were found between GDF15 and ferritin as well as LDH level.^17^ In contrast, Shokrgozar and colleagues found no significant correlation between the GDF15 and serum ferritin levels.^14^ Our results were in agreement with Ozturk and colleague as they found significantly positive correlation between GDF15 level and ERFE, and no significantly correlations with Hb%, ferritin, or hepcidin levels.^21^ The aforementioned findings could be explained by the fact that in these patients, the ineffective erythropoiesis with the resultant anemia, and hypoxemia lead to increased secretion of erythropoietin, as well as reduced levels of hepcidin with subsequent increased intestinal iron absorption and iron release from its stores in the macrophages.^22^

The variability in the relationship between GDF15 levels and other parameters (for example; Hb, hepcidin, and ferritin) could be explained by the different regimens used to manage thalassemia patients, as per a study that found lower serum GDF15 level in thalassemic patients on proper chelation regimens and in those on hydroxyurea therapy, probably due to the decreased ineffective erythropoiesis.^17^ As our patients are generally under-transfused (pretransfusion level is less than 8 g/dl), higher erythroid activity and subsequent ineffective erythropoiesis are prevalent. Ineffective erythropoiesis may produce signals that increase GDF15 gene expression.^16^

**Fig. 1:**
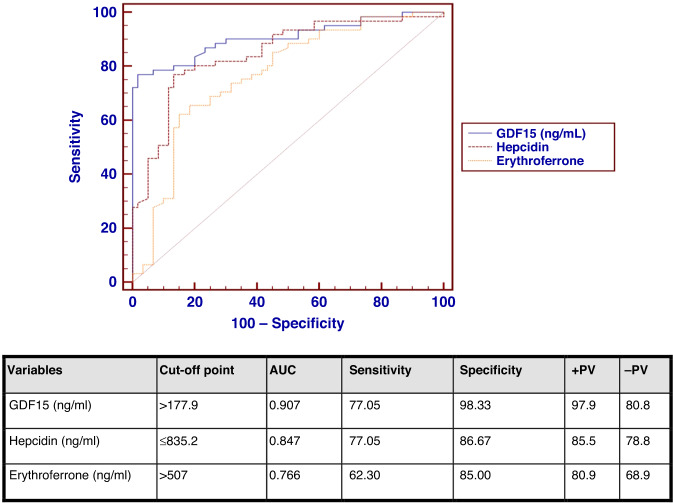
ROC curve analysis for the studied variables. ROC curve analysis for GDF15, hepcidin, and erythroferrone. Sensitivity and specificity for detection of iron overload.

We used a cut-off point of >177.9 ng/ml with area under the curve (AUC) of 0.907 with a sensitivity of 77.05% and specificity of 98.33% in detection of iron overload (Fig. 1). To our knowledge, the predictability of iron overload using serum levels of GDF15 has not been researched before.

### Regarding ERFE

In our study, ERFE was significantly higher in patients than controls (medians were 992.4 and 159.6 ng/ml respectively) with a *P* value < 0.001. This was in agreement with another study that confirmed higher levels of ERFE in thalassemic patients.^23^

ERFE play a crucial role in hepcidin inhibition and iron redistribution after hemolytic attacks, its sustained elevation is involved in iron overload.^24^ We found that patients with higher ferritin levels had significantly higher levels of ERFE (*P* value < 0.001). This finding was explained by the persistently active erythropoietic drive in thalassemia patients with the resultant hepcidin suppression through sustained stimulation of ERFE production. The resultant hypoxia further augments this effect. Eventually, both (ERFE and hypoxia) will result in iron overload.^25^

This study showed that ERFE had highly significant negative correlation with Hb, a significantly negative correlation with hepcidin, highly significant positive correlations with GDF15, reticulocyte count, LDH level, and serum ferritin. These findings were in agreement with a study by Almousawi and Sharaba regarding the negative correlation with hemoglobin, while in disagreement with their results regarding serum ferritin, where they found a negative correlation between ERFE and ferritin.^26^ Similar to ours, Saad and colleagues found a significantly positive correlation between ERFE and ferritin; however, there was no correlation with Hb or hepcidin.^23^

Regarding the ROC curve for ERFE, the cut-off point of >507 ng/ml, and the AUC of 0.766 were selected in our study to detect iron overload with 62.30 % sensitivity and 85% specificity (Fig. 1). On another study a cut-off point of >450 ng/ml and AUC of 0.806 were selected with 91.6% sensitivity and 65% specificity in detecting iron overload.^10^

### Hepcidin

Serum hepcidin was significantly lower in the thalassemia group compared to the control group (means= 535.7 ng/ml and 2130 ng/ml, respectively) with *P* value < 0.001. Similar results were found by other researchers.^23^ Several studies declared that the peripheral expression of hepcidin messenger RNA was downregulated among β thalassemia patients, compared to healthy controls.^25,27^ This could be explained by the effect of chronic hypoxia, caused by ineffective erythropoiesis that acts mainly through hypoxia-inducible factor-2α (HIF-2α), which increases the expression of divalent metal transporter 1, duodenal cytochrome B, and ferroprotein, resulting in hepcidin suppression, which eventually increases both serum heme and non-heme iron.^25,27^ Contrary to our results, similar levels of serum hepcidin in β-thalassemia patients and normal controls were reported in another study.^28^ These differences can be explained by the presence of multiple factors, such as proper chelation and transfusion regimen, accounting for inter-individual variation.

This study showed that serum hepcidin level had highly significant negative correlations with GDF15, reticulocyte count, LDH level, and serum ferritin. Also significantly negative correlations with ERFE, while it had highly significant positive correlation with Hb. A previous study found positive correlation between hepcidin and Hb level.^29^ Several other studies failed to detect any correlation between serum hepcidin and ERFE,^23^ ferritin levels,^10,23,25,27^ and Hb%.^10,25,27^

Regarding the ROC curve for hepcidin to assess iron overload, a cut-off point of <835.2 ng/ml and AUC of 0.847 were selected with 77.05% sensitivity and 86.67% specificity (Fig. 1). Ismail and his colleagues previously found that hepcidin has a significant predictability of iron overload with the AUC of 0.628.^30^

Several clinical trials are currently in various phases of development to study the use of GDF15 inhibitors in a variety of diseases like prostate cancer,^31^ melanoma,^32^ obesity,^33^ and cardiac infarction.^34^ The current study might motivate researchers to study the effect of GDF15 antagonist in the prevention and management of iron overload.

## Conclusion and recommendation

We concluded that in conditions with ineffective erythropoiesis, the elevated GDF15 values together with elevated ERFE level could be partially responsible for the pathological iron burden, through inhibition of the serum hepcidin level. Also, serum ERFE, GDF15, and hepcidin could be used as biomarkers and indicators of iron overload in patients with beta-thalassemia. We recommend more studies to be done in order to assess GDF15 and ERFE as new biomarkers of iron overload and to start the assessment of the potential therapeutic agents targeting these biomarkers in patients with β thalassemia syndromes. We also recommend conducting longitudinal studies to investigate the impact of GDF15 polymorphism on the variable response and possible resistance to iron chelators, and also on the risk of development of cardiac and endocrine complications in thalassemia patients

### Limitations

Our study had some limitations; for starter, the relatively small numbers of patients compared to the prevalence of thalassemia in our country, secondly, the lack of MRI iron study, which is a more accurate tool for estimation of tissue iron overload than serum ferritin, and lastly, the cross-sectional nature of the study, which rendered us unable to correlate the presence of GDF15 gene polymorphism and high GDF15 level to other associated disorders, like resistance to iron chelators and development of endocrinal disorders.

## Data Availability

Upon request to the corresponding author. all data, from which the conclusions of our paper were made, will be freely available to fellow researcher wishing to use them for non-commercial purposes.
